# Evaluation of the Standard M10 MTB/NTM Molecular Test for the Rapid Identification of Tuberculous and Nontuberculous Mycobacteria in Liquid Cultures

**DOI:** 10.3390/pathogens14060517

**Published:** 2025-05-22

**Authors:** Sara Caldrer, Alberta Carrara, Andrea Ragusa, Lavinia Nicolini, Elena Pomari, Cristina Mazzi, Fabio Formenti, Francesca Perandin

**Affiliations:** 1 Department of Infectious—Tropical Diseases and Microbiology, IRCCS Sacro Cuore Don Calabria Hospital, 37024 Verona, Italyelena.pomari@sacrocuore.it (E.P.);; 2 Centre for Clinical Research, IRCCS Sacro Cuore Don Calabria Hospital, 37024 Verona, Italy

**Keywords:** *Mycobacterium tuberculosis*, non-tuberculous mycobacteria, MGIT culture, real-time PCR, Sanger sequencing, POCT, non-endemic areas

## Abstract

Since 2013, the World Health Organization has recommended the use of rapid molecular tests as the initial diagnostic step for *Mycobacterium tuberculosis* (MTB) infection to enhance the control of tuberculosis. In recent years, the prevalence of infections by non-tuberculous mycobacteria (NTM) in humans has also risen, particularly in countries with low tuberculosis incidence, such as Italy. Therefore, the rapid differentiation between NTM and *Mycobacterium tuberculosis* complex is crucial for timely therapeutic decisions. This study evaluates a new rapid molecular assay, Standard M10 MTB/NTM, designed to detect MTB, NTM, or co-detection in Mycobacteria Growth Indicator Tube cultures from different biological matrices. The assay was validated using 100 positive and 50 negative liquid mycobacteria cultures, already confirmed by specific real-time PCR and Sanger sequencing. Following optimization of assay conditions for culture sample processing and assessment of potential interference, Standard M10 demonstrated excellent sample stability, high specificity, and good sensitivity, identifying all 50 MTB and 49 NTM samples. Some limitations included the non-detection of *M. celatum* in one case and false positive results (MTB co-infection) in two NTB cases. Nevertheless, overall, the adoption of this test could be considered for laboratory management to enable rapid and effective sample targeting for subsequent diagnostic evaluation and treatment decision-making.

## 1. Introduction

Human tuberculosis (TB), caused mainly by *Mycobacterium tuberculosis* (MTB), remains a major public health concern, with an estimated 10.6 million new cases and 1.3 million deaths reported worldwide in 2022 [1]. In recent years, there has been a notable increase in the number of diagnoses of non-tuberculous mycobacteria (NTM) infections, likely due to advances in diagnostic techniques and increased awareness of the condition among healthcare professionals [2,3]. NTM are environmental organisms found in water and soil and include over 170 different species with a geographically heterogeneous location; some species are implicated worldwide (i.e., *M. avium* complex), and others are regionally significant (*M. malmoense*) [4]. NTM cause a spectrum of diseases that include tuberculosis (TB)-like pulmonary and extrapulmonary disease, cervical lymphadenitis in young children, and visceral and disseminated disease [5]. NTM are capable of causing infections, particularly in individuals with underlying conditions such as immunodeficiency, cystic fibrosis, or chronic obstructive pulmonary disease [6].

In Italy, the prevalence of NTM infections is estimated to range from 1.0 to 1.8 cases per 100,000 inhabitants, with notable regional variations [7]. A 2016 study reported a prevalence of 6.2 cases per 100,000 inhabitants for NTM lung disease (NTM-LD) in Italy, consistent with observations in other European countries (e.g., the UK, Spain, France, and Germany) [8]. In countries with a low TB incidence, such as Italy, a diagnostic approach that allows for the rapid distinguishing between NTM and *Mycobacterium tuberculosis* complex (MTBC) is essential for the effective management of mycobacterial diseases, enabling timely and targeted therapeutic decisions [9]. As outlined in the survey conducted by Stroffolini [10], molecular tests that allow NTM identification are available in 40% of the participating centers in Italy. Concurrently, phenotypic tests are available only in 30% of these centers, with a notable concentration of this located in the northern region, encompassing university hospitals and outpatient clinics [10].

To date, diagnostic strategies have predominantly focused on MTB, while NTM detection has typically relied on culture testing based on clinical suspicion. For primary samples, such as sputum, the World Health Organization (WHO) recommends using the Xpert MTB/RIF system (Cepheid, Sunnyvale, CA, USA) for TB diagnosis, which makes specific TB diagnoses and can also detect resistance to rifampicin [5] but is unable to detect NTM infection. Moreover, the WHO suggested the use of rapid immunochromatographic tests (ICTs) based on a predominant protein, MPT64, secreted by the MTBC for their identification in positive cultures [11]. The latter are frequently included in the diagnostic algorithm for rapid indirect diagnosis (through the exclusion of MTB infection) in a Mycobacteria Growth Indicator Tube (MGIT) or microscope-positive specimens, especially in developing countries, due to rapid results and technical simplicity [12]. Even in this case, NTM identification is hampered by the absence of rapid methodologies for NTM detection in culture samples.

The Standard M10 molecular diagnostic system (SD Biosensor Inc., Suwon, Republic of Korea) was introduced in 2020 and constitutes a simple and rapid random-access cartridge-based PCR system with a point-of-care design, highly analogous to the GeneXpert system [13]. The system includes two CE-certified (Conformité Européenne) in vitro diagnostic (IVD) assays for the rapid detection of MTB in human normal sputum or a sputum sediment specimen: the M10 MDR-TB (for MTB detection and the identification of drug resistance) and the MTB/NTM assay that simultaneously detects MTB and NTM. MTB detection is based on the IS6110 insertion element, whereas NTM detection is based on the pan-mycobacterial internal transcribed spacer (ITS) region.

A previous study evaluated both the Standard M10 MDR-TB and the MTB/NTM on primary samples in a low-tuberculosis setting, comparing them with the Xpert MTB/RIF Ultra test. The results demonstrated that the M10 MDR-TB exhibited a sensitivity of 88.9% and a specificity of 97.4% in the detection of MTB, while the M10 MTB/NTM demonstrated a sensitivity and a specificity of 65.7% (CI = 49.1–79.2%) and 96.8% (CI = 91.8–99.0%) and exhibited certain limitations in the detection of NTM [14,15].

The present study aims to evaluate the diagnostic performance of the M10 MTB/NTM rapid molecular test to detect the presence of MTB, NTM, or a co-infection (MTB/NTM) in MGIT culture samples obtained from different biological matrices that have been previously characterized at the genomic identification level for NTM. Furthermore, the comparison with the rapid immunochromatographic test on a lateral flow card (SD Biosensor Standard Q MTP64—SD Biosensor Inc., Suwon, Republic of Korea), designed to detect the MTP64 MTB antigen using MGIT medium samples, was performed. The experimental plan was designed to evaluate the potential of the SD system as a valuable diagnostic tool for rapid differentiation between NTM and MTB in a low-endemic TB laboratory setting in order to enhance the effective and timely management of mycobacterial diseases.

## 2. Materials and Methods

### 2.1. Clinical Samples

This study was conducted using mycobacterial liquid cultures previously frozen in glycerol solution and stored in the strain library at the Tropica Biobank (bbmri-eric:ID:IT_1605519998080235), Department of Infectious—Tropical Diseases and Microbiology of IRCCS Sacro Cuore Don Calabria Hospital. The MGIT cultures were obtained from different clinical isolates, as described in Table 1. A total of 100 mycobacteria culture-positive samples were tested, 50 positive for MTB and 50 for NTM species. Moreover, 50 MGIT negative cultures were used as the controls (Table 1). The MTB positivity was simultaneously confirmed using a commercial real-time PCR kit (MDR/MTB ELITe MGB^®^ Kit, ELITechGroup, Turin, Italy), while the NTM identification was performed by Sanger sequencing (Table 2).

All MGIT cultures were thawed and re-cultured prior to experimental procedures. Sample processing, like sample pre-treatment, was performed in a Class III Biosafety Cabinet. Meanwhile, the real-time PCR procedure was performed in Class II laboratory conditions [16].

### 2.2. All-in-One Mycobacteria Real-Time PCR System

The molecular diagnostic test used in this study was the SD Biosensor Standard M10 MTB/NTM kit (SD Biosensor Standard M10 MTB/NTM—SD Biosensor Inc., Suwon, Republic of Korea), validated by the manufacturer from human normal sputum or sputum sediment specimens. It is an all-in-one cartridge (extraction/amplification) for the SD Biosensor M10 instrument, using the pre-treatment (sample reagent) supplied with the kit.

Before the experimental phase, an optimisation process was undertaken to set working conditions according to several parameters.

(1) Stability time was determined by evaluating the reliability of the MTB/NTM culture sample treated with the sample reagent (SR) at a 1:3 dilution (as recommended by the manufacturer [17]) at various times (from 15 min to 4 h) before performing real-time PCR using the MTB/NTM cartridge. The manufacturer suggested an incubation time of about 15 min.
(2) The culture dilution process is a crucial aspect of the overall methodology. The manufacturer’s recommended sample dilution was 1:3 with the sample reagent when applied to sputum. To assess any potential loss of sensitivity in the culture setting, higher dilutions of the MGIT culture were performed, ranging from 1:2 to 1:16.
(3) The impact of mixed strains on interferences and sensitivity was investigated. The specificity of the SD Biosensor Standard M10 MTB/NTM Kit (SD Biosensor Inc., Suwon, Republic of Korea) was examined by creating mixed cultures (MTB/NTM ratio 4:8–4:16, and vice versa) to confirm the system’s capacity to detect these cultures appropriately.
(4) The safety of operators was assessed by evaluating the inactivating capacity of the solubilizing reagent against mycobacterial cultures. A portion of the aliquot treated with the sample reagent was re-cultured to ascertain the inactivating capacity of the reagent.

### 2.3. Immunochromatography Test for MPT64 Antigen Detection

The immunochromatography reference kit, Standard Q TB MPT64 Ag (SD Biosensor Inc., Suwon, Republic of Korea), was used according to the manufacturer’s guidelines. This evaluation method is designed for the qualitative analysis of the MTB MPT64 antigen in solid or liquid culture specimens through the use of a straightforward methodology that enables the acquisition of screening test results within a timeframe of 10 min. We concurrently performed this test on approximately 200 µL of liquid cultures.

### 2.4. Statistical Analysis

For MTB/NTM DNA detection in samples, the sensitivity and specificity were calculated using the liquid culture stored in the strain library at the Tropica Biobank as a reference. Descriptive statistics were computed using Microsoft Excel (Microsoft, Redmond, Washington, DC, USA). Furthermore, 95% confidence intervals (CIs) were determined using the Wilson score with the continuity correction method using R software version 4.4.2 (RCore Team, Vienna, Austria). Graphical representations were generated with GraphPad Prism 10.2.0 (GraphPad Software, San Diego, CA, USA).

## 3. Results

### 3.1. Preliminary Results Described Good Specificity and Stability of the SD Biosensor Standard M10 MTB/NTM Test on MGIT Culture

The optimisation revealed that the stability of the sample following treatment with SR was maintained for up to 4 h, without any significant decline in genomic detection (expressed as Ct), as illustrated in Table 3. The stability of the internal control (IC) lends further support to the conclusion that the test is optimized to a satisfactory degree and can be performed over a fairly wide time interval without compromising the results. This is a particularly important consideration in a laboratory that processes a high volume of samples daily, as it allows a longer time window to process and analyze the samples without compromising sensitivity.

The manufacturer’s recommendations specify that the sample reagent should be used at a 1:3 dilution when applied to sputum. Accordingly, with the higher mycobacteria concentration in liquid cultures, a sample serial dilution was performed with SR, using a culture ratio ranging from 1:2 to 1:16. As outlined in Table 4, no substantial reduction in bacterial load (expressed as Ct) was observed. In detail, a reduction of 2 Ct linked to an 8-fold dilution was measured. This finding suggests that the SD Biosensor Standard M10 MTB/NTM test remains sensitive, even when the sample is subjected to high dilution.

Furthermore, test specificity was assessed by creating mixed cultures (different MTB:NTM ratios) and verifying whether the system could detect these cultures appropriately. As described in Table 5, MTB Ct values showed good stability with minimal variation between ratios (Ct within 20.45 to 22.41; Delta Ct < 2), suggesting a consistent sensitivity for MTB. In contrast, the Ct values for NTM exhibited slightly higher variability (Ct increased from 19.57 to 21.18; Delta Ct > 2), suggesting that the test may be more susceptible to changes in NTM than MTB rates, according to previous observations [14].

Finally, the inactivation capacity of SR against mycobacterial cultures was evaluated to assess operator safety. Samples re-cultured after SR treatment became positive within one day, indicating that SR does not effectively inactivate mycobacteria. This finding is crucial for laboratory workflow management, highlighting the need for sample culture handling in a Biosafety Level 3 (BSL-3) laboratory.

### 3.2. The SD Biosensor Standard M10 MTB/NTM Test Shows High Specificity for MTB on MGIT Culture

A total of 100 mycobacteria culture-positive samples were tested, 50 samples positive for MTB and 50 for NTM species (previously identified by Sanger sequencing); moreover, 50 MGIT-negative cultures were used as the negative controls. Based on the previous stability tests, experiments were conducted under the following conditions: RT-PCR tests with SD Biosensor Standard M10 MTB/NTM were performed one hour after the addition of a sample reagent at a 1:4 ratio.

**Figure 1:**
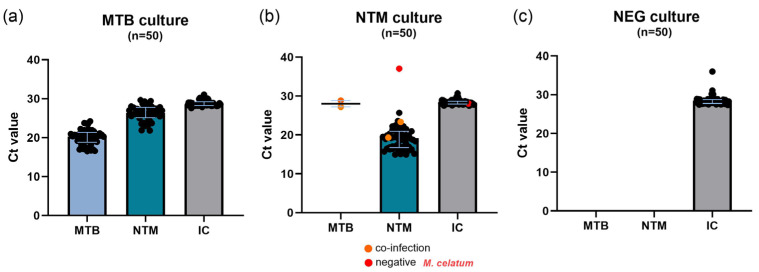
Bar plots representing the Ct values obtained after the MTB/NTM-PCR performed on positive MGIT culture separated based on the specific probe for MTB, NTM, and the IC. (**a**) Bar plot represents the mean and SD of Ct values obtained after SD Biosensor Standard M10 MTB/NTM test performed on MTB-positive MGIT cultures. (**b**) Bar plot represents the mean and SD of Ct values obtained after SD Biosensor Standard M10 MTB/NTM test performed on NTM-positive MGIT cultures. (**c**) Bar plot represents the mean and SD of Ct values obtained after SD Biosensor Standard M10 MTB/NTM assay performed on negative MGIT culture.

The M10 MTB/NTM system demonstrated a good performance in MTB detection, as described in Figure 1a and Table S1. All samples resulted in being positive for MTB, with relatively low Ct values (mean 20.03, SD 1.9), indicating an optimal load of MTB in the samples. In contrast, the NTM signal, although detected, was measured at higher Ct values (mean 26.20, SD 1.9). The test algorithm defined this result as the absence of NTM in the sample. The Ct values for MTB and NTM measured a mean difference of approximately 6.17 (SD 0.87). Furthermore, the mean Ct value for the internal control was 28.81 (SD 0.76), indicating that the test was performed correctly in all samples. The assay’s performance was confirmed by calculating the sensitivity and specificity at 100% (95% CI: 91.1–100) for both gene targets (IS6110 and ITS).

By evaluating the results obtained by NTM culture analysis with an SD Biosensor Standard M10 MTB/NTM test, we observed that 47/50 samples resulted as being positive for NTM, with Ct values relatively low (19.37 mean, 3.7 SD), and the absence of MTB (as expected by algorithm), as shown in Figure 1b and Table S2. In two cases, the SD Biosensor Standard M10 MTB/NTM test detected both MTB and NTM mycobacteria. The first case was a culture of *M. avium* in which the system detected both the MTB genome (Ct 24.91) and the NTM genome (Ct 19.33); the second was a culture of *M. chimaera* in which the system detected the MTB genome (Ct 27.2) and the NTM genome (Ct 23.36), always respecting the NTM > MTB Ct ratio. Moreover, the assay was not able to indicate positivity for a culture of *M. celatum* despite being able to identify genomic material for the NTM probe (Ct 37.04). The mean Ct value for the internal control was 27.83 (4 SD), indicating that the assays performed well. Based on these results, we calculated the sensitivity and specificity for both genetic targets. The sensitivity and specificity were 96.0% (95% CI: 85.1–99.3) and 98.0% (95% CI: 88.0–99.9), respectively.

Finally, the M10 MTB/NTM system demonstrated a specificity of 100% (95% CI: 91.1–100), as described in Figure 1c and Table S3. All 50 negative MGIT cultures were negative for both MTB and NTM probes. In this contest, we only analyzed negative MGIT cultures and not random bacterial cultures (such as *Nocardia* spp.) to assess the test specificity, which should be considered in the future for further validation of the assay.

### 3.3. The Antigenic Test Correctly Identified the MTB Culture

Finally, we evaluated the performance of an immunochromatographic test (Standard Q TB MPT64 Ag) designed to detect MTBC in liquid cultures and compared the results with those obtained using the SD Biosensor Standard M10 MTB/NTM test. The ICT test for MPT64 antigen detection successfully identified *M. tuberculosis* in all of the MTB-positive cultures and without positives in NTM and negative MGIT cultures.

## 4. Discussion

This study evaluated the diagnostic performance of the Standard M10 MTB/NTM molecular assay for detecting MTB, NTM, or co-infections in previously characterized MGIT cultures. The primary objective was to assess its diagnostic utility for rapid differentiation between NTM and MTB in a low-endemic TB laboratory setting, aiming to improve the effective and timely management of mycobacterial diseases.

This approach is particularly beneficial for healthcare facilities that lack the necessary technical expertise or equipment to accurately identify NTMs. As a result, these centers frequently refer specimens to a reference centre for species identification. The use of a molecular system capable of rapidly detecting NTMs in specimens has the potential to streamline both sample and patient management.

This study was prompted by the increase in non-tuberculous mycobacterial infections diagnosed at our hospital in recent years, which requires a more rapid diagnostic response. Additionally, the limitations of existing diagnostic strategies for the rapid identification of NTMs, unlike MTB, typically rely on culture examination based on clinical suspicion.

As previously discussed, the two main WHO-recommended tests for the rapid identification of mycobacteria are predominantly focused on MTB detection [5], and neither is able to detect NTM infection. Furthermore, only a limited number of commercial tests are currently available that enable the simultaneous identification of both tuberculous and non-tuberculous mycobacteria (such as Anyplex™ MTB/NTM Real-time Detection, Seengenes, or a GENEDIA MTB/NTM Detection Kit). These are real-time polymerase chain reaction (RT-PCR) tests that can be performed manually in laboratories equipped with the appropriate instrumentation, in contrast to point-of-care tests (POCTs), which are designed for ease of use, even in resource-limited laboratory settings. Therefore, given the low TB incidence of tuberculosis in Italy, the ability to efficiently differentiate between tuberculous and non-tuberculous mycobacterial infection has become an important and valuable diagnostic tool for effective clinical management.

The Standard M10 MTB/NTM assays closely resemble the Xpert MTB assays [15]. The workflow involves an initial chemical pre-treatment step followed by sample transfer into a self-contained assay cartridge. The cartridge is then loaded into a scalable, random-access system comprising a system unit and independently operating analyzer modules. The most noticeable difference between the assays, besides NTM detection, is the inclusion of an additional syringe filtration step in the Standard M10 pre-treatment protocol and sample reagent composition [17]. The Xpert Sample Reagent has been shown to reduce viable MTB by more than 6 log units and eliminate growth in most MTB-positive sputum samples [18]. In contrast, the Standard M10 pre-treatment allowed the growth of MTB, similar to the samples without pre-treatment. Similar to Xpert MTB, the Standard M10 MTB/NTM assays provide results in about 70 min (faster than the 90 min required for Xpert MTB).

Our results suggested that the Standard M10 MTB/NTM assay offered several advantages. First, sample stability following treatment with the sample reagent (SR) was maintained for up to four hours, without any significant loss in detection sensitivity, as evidenced by consistent Ct values (as shown in Table 3); this is a pivotal consideration in laboratory management. Additionally, as outlined in Table 4, a non-significant reduction in bacterial load was observed after sample dilution, suggesting high performance and sensitivity, even when the sample is subjected to significant dilution. Considering the interferences and sensitivity in the presence of mixed strains, it was observed that the Standard M10 MTB/NTM assay was capable of detecting these cultures appropriately, demonstrating notable stability and exhibiting minimal variation across different ratios (as represented in Table 5). Finally, the M10 MTB/NTM assay has shown good performance in MTB/NTM detection (as described in Figure 1). The assay demonstrated sensitivity and specificity at 100% (95% CI: 91.1–100) for MTB and 96.0% specificity (95% CI: 85.1–99.3) and 98.0% sensitivity (95% CI: 88.0–99.9) for NTM samples and was capable of detecting over 11 distinct species of NTMs.

Conversely, the Standard M10 MTB/NTM test exhibited some limitations in analytical specificity and reactivity. Notably, two NTM clinical cultures—one identified as *M. chimaera* and the other as *M. avium*—were misclassified as MTB-NTM “co-infection”, as described in Figure 1b. In contrast, the ICT test did not yield any false-positive results for MTB. The potential implications of false-positive MTB test results are clinically significant, as they may lead to unnecessary drug regimens and patient isolation. Recently, the manufacturer released an M10 software upgrade version, which should be able to avoid misclassified coinfections. Additionally, the inability to detect all NTM species, such as the presence of *M. celatum* in a culture, despite the identification of NTM probe genomic material, is a noteworthy limitation. Finally, a positive NTM-PCR result necessitates further species-level identification to guide appropriate therapeutic management. Therefore, to comprehensively evaluate the clinical performance and utility of the Standard M10 MTB/NTM test in various demographic groups and patient environments, several research and validation initiatives need to be undertaken.

## 5. Conclusions

The Standard M10 MTB/NTM test has demonstrated good specificity for detecting MTB and NTM in culture specimens, with stability over time and a rapid response. However, its suitability may be limited by biosafety concerns due to the weak inactivation of MTB during sample pre-treatment. Additionally, identifying the NTM species is crucial, as targeted therapeutic measures cannot be implemented without it. In conclusion, this test may be particularly useful in clinical settings characterized by a low incidence of TB in centers with limited capacity for NTM identification, helping to optimize sample management and efficiently direct positive samples to the reference centre for further testing.

## Figures and Tables

**Table 1:** Clinical isolates on which mycobacteria were detected (*n* = 150).

Biological Matrices	NTM, *n* = 50	MTB, *n* = 50	NEG, *n* = 50
Bronchial aspirate	21 (42%)	2 (4.0%)	21 (42%)
Sputum	12 (24%)	32 (64.0%)	10 (20%)
Bronchial lavage	16 (32%)	4 (8.0%)	18 (36%)
Biopsy	1 (2%)	6 (12.0%)	0 (0.0%)
Urine	0 (0.0%)	4 (8.0%)	0 (0.0%)
Lymph node aspirate	0 (0.0%)	0 (0.0%)	1 (2.0%)
Cerebrospinal fluid	0 (0.0%)	1 (2.0%)	0 (0.0%)
Pleural fluid	0 (0.0%)	1 (2.0%)	0 (0.0%)

**Table 2:** NTM species identified by Sanger sequencing (*n* = 50).

NTM Strains	Freqs (% of Total)	
*Mycobaterium avium* complex	21	(42%)
*Mycobaterium intracellulare*	7	(14%)
*Mycobaterium gordonae*	6	(12%)
*Mycobacteriodes abscessus*	4	(8%)
*Mycobaterium chimaera*	4	(8%)
*Mycolicibacterium fortuitum*	2	(4%)
*Mycobacterium intracellulare* sub. *M. chimaera*	1	(2%)
*Mycobaterium xenopi*	1	(2%)
*Mycobacteriodes chelonae*	1	(2%)
*Mycolicibacterium mucogenicum*	1	(2%)
*Mycobaterium simiae*	1	(2%)
*Mycobaterium celatum*	1	(2%)

**Table 3:** Stability test after sample-reagent pre-treatment.

Time	Ct MTB	Ct NTM	Ct IC
15 min *	neg	19.76	26.85
1 h	neg	19.87	27.59
2 h	neg	19.77	27.20
3 h	neg	19.71	27.86
4 h	neg	19.43	27.32

* Condition suggested by manufacturer.

**Table 4:** Stability test after sample-reagent dilution.

Sample:Reagent Ratio	Ct MTB	Ct NTM	Ct IC
1:2	neg	21.39	28.26
1:4	neg	23.03	29.70
1:8	neg	22.38	27.33
1:16	neg	23.30	27.55

1:3 condition suggested by manufacturer.

**Table 5:** Interference and sensitivity in the presence of mixed strains.

MTB:NTM Ratio	Ct MTB	Ct NTM	Ct IC
8:4	21.38	19.57	27.03
4:8	21.37	20.86	27.37
4:16	20.54	21.18	27.28
16:4	22.41	19.02	27.18

## Data Availability

Row data are present in Supplementary Materials.

## Supplementary Materials

The following supporting information can be downloaded at: https://www.mdpi.com/article/10.3390/pathogens14060517/s1, Table S1: Descriptive data of diagnostic test results obtained from MTB cultures; Table S2: Descriptive data of diagnostic test results obtained from NTM cultures; Table S3: Descriptive data of diagnostic test results obtained from negative MTIG cultures.

## Abbreviations

The following abbreviations are used in this manuscript:

MTBC	*Mycobacterium tuberculosis* complex
MTB	*Mycobacterium tuberculosis*
NTM	Non-Tuberculous Mycobacteria
MGIT	Mycobacteria Growth Indicator Tube
PCR	Polymerase Chain Reaction
WHO	World Health Organization
POCT	Point Of Care Test
ICTs	Immunochromatography Tests
TB	Tuberculosis
RIF	Rifampicin
Ct	Cycle Threshold
BSL3	BioSafety Level 3 Laboratory
SD	Standard Deviation
CIs	Confidence Intervals

## References

[1] Tobin E.H., Tristram D. (2025). Tuberculosis Overview. StatPearls.

[2] Bethencourt Mirabal A., Nguyen A.D., Ferrer G. (2025). Lung Nontuberculous Mycobacterial Infections. StatPearls.

[3] Loebinger M.R., Welte T. (2016). Current Perspectives in the Diagnosis and Treatment of Nontuberculous Mycobacterial Pulmonary Disease. Eur. Respir. Pulm. Dis..

[4] Jing H., Wang H., Wang Y., Deng Y., Li X., Liu Z., Graviss E.A., Ma X. (2012). Prevalence of Nontuberculous Mycobacteria Infection, China, 2004–2009. Emerg. Infect. Dis..

[5] Gopalaswamy R., Shanmugam S., Mondal R., Subbian S. (2020). Of Tuberculosis and Non-Tuberculous Mycobacterial Infections—A Comparative Analysis of Epidemiology, Diagnosis and Treatment. J. Biomed. Sci..

[6] Kumar K., Ponnuswamy A., Capstick T.G., Chen C., McCabe D., Hurst R., Morrison L., Moore F., Gallardo M., Keane J. (2024). Non-Tuberculous Mycobacterial Pulmonary Disease (NTM-PD): Epidemiology, Diagnosis and Multidisciplinary Management. Clin. Med..

[7] Aliberti S., Codecasa L.R., Gori A., Sotgiu G., Spotti M., Di Biagio A., Calcagno A., Nardini S., Assael B.M., Tortoli E. (2018). The Italian Registry of Pulmonary Non-Tuberculous Mycobacteria—IRENE: The Study Protocol. Multidiscip. Respir. Med..

[8] Giannoni F., Lanni A., Iacobino A., Fattorini L., Italian Multicentre Study on Nontuberculous Mycobacteria (IMS-NTM), members of the IMS-NTM laboratory network (2023). Epidemiology and Drug Susceptibility of Nontuberculous Mycobacteria (NTM) in Italy in 2016–2020. Ann. Ist. Super. Sanita.

[9] Sharma S.K., Upadhyay V. (2020). Epidemiology, Diagnosis & Treatment of Non-Tuberculous Mycobacterial Diseases. Indian J. Med. Res..

[10] Stroffolini G., Lupia T., Gaviraghi A., Venuti F., Cinnirella G., Gori A., Spotti M., Blasi F., Codecasa L., Calcagno A. (2025). Prescription Habits and Drugs Accessibility for the Treatment of Non-Tuberculous Mycobacteria Infections in Italy: A Multicentric Survey from the IRENE Study Group. Infection.

[11] Phunpae P., Thongkum W., Panyasit W., Laopajon W., Takheaw N., Pata S., Yasamut U., Kasinrerk W., Tayapiwatana C. (2024). Rapid Lateral Flow Test for *Mycobacterium tuberculosis* Complex and Non-Tuberculous Mycobacteria Differentiation. Appl. Microbiol. Biotechnol..

[12] Lwoga E.T., Sangeda R.Z. (2019). ICTs and Development in Developing Countries: A Systematic Review of Reviews. Electron. J. Inf. Syst. Dev. Ctries..

[13] Abdullah A., Sam I.-C., Ong Y.J., Theo C.H., Pukhari M.H., Chan Y.F. (2023). Comparative Evaluation of a Standard M10 Assay with Xpert Xpress for the Rapid Molecular Diagnosis of SARS-CoV-2, Influenza A/B Virus, and Respiratory Syncytial Virus. Diagnostics.

[14] Luukinen B., Aittoniemi J., Miikkulainen-Lahti T., Mentula S., Pätäri-Sampo A. (2024). Evaluation of the STANDARD M10 MDR-TB and MTB/NTM Assays for the Detection of Mycobacterium Tuberculosis, Rifampicin and Isoniazid Resistance, and Nontuberculous Mycobacteria in a Low-Incidence Setting. J. Clin. Microbiol..

[15] Stephen S., Kadye A., Majuru X.N., Madamombe T., Sokwe J., Madondo T., Tinarwo K., Tsuvani L., Kawome T., Malunga F. (2024). Diagnostic Performance of STANDARD^TM^ M10 Multidrug-Resistant Tuberculosis Assay for Detection of *Mycobacterium tuberculosis* and Rifampicin and Isoniazid Resistance in Zimbabwe. Int. J. Mycobacteriology.

[16] World Health Organization (2012). Essential biosafety measures for TB laboratories. Tuberculosis Laboratory Biosafety Manual.

[17] STANDARDTM M10 MTB/NTM Manufacturer’s Recommendations. https://www.relabsrl.it/product/standard-m10-mtb-ntm/.

[18] Banada P.P., Sivasubramani S.K., Blakemore R., Boehme C., Perkins M.D., Fennelly K., Alland D. (2010). Containment of Bioaerosol Infection Risk by the Xpert MTB/RIF Assay and Its Applicability to Point-of-Care Settings. J. Clin. Microbiol..

