# Supplementary material for: Evaluation of the Standard M10 MTB/NTM Molecular Test for the Rapid Identification of Tuberculous and Nontuberculous Mycobacteria in Liquid Cultures
Source: Pathogens. 2025 May 22;14(6):517. doi: 10.3390/pathogens14060517 (PMC12195744; doi:10.3390/pathogens14060517)
Supplement: Supplementary file 1 [file pathogens-14-00517-s001.zip › pathogens-3631074-supplementary.pdf]

Supplementary Tables

Table S1: Descriptive data of diagnostic test results obtained from MTB cultures

CULTURE MATRIX	SD BIOSENOR RESULT	MTB Ct	NTM Ct	IC Ct	STANDARD Q TB MPT64 Ag	Δ Ct (NTM-MTB)
Sputum	**MTB**	18.26	23.43	28.58	Positive	5.17
Sputum	**MTB**	19.65	25.82	28.87	Positive	6.17
Sputum	**MTB**	19.30	25.48	28.49	Positive	6.18
Biopsy	**MTB**	21.76	28.21	30.20	Positive	6.45
BLav	**MTB**	16.96	23.73	28.17	Positive	6.77
Biopsy	**MTB**	20.17	25.83	28.39	Positive	5.66
Sputum	**MTB**	20.26	26.94	28.35	Positive	6.68
Sputum	**MTB**	20.70	28.00	29.01	Positive	7.3
Biopsy	**MTB**	23.65	28.83	29.87	Positive	5.18
BAsp	**MTB**	18.59	24.99	28.14	Positive	6.4
PF	**MTB**	20.30	26.45	28.14	Positive	6.15
Sputum	**MTB**	19.49	25.19	29.09	Positive	5.7
Biopsy	**MTB**	17.4	23.54	28.17	Positive	6.14
Sputum	**MTB**	18.93	26.33	28.30	Positive	7.4
CFS	**MTB**	17.09	23.04	28.86	Positive	5.95
Urine	**MTB**	20.48	26.65	30.12	Positive	6.17
Sputum	**MTB**	20.38	26.73	28.25	Positive	6.35
Sputum	**MTB**	18.26	24.43	28.28	Positive	6.17
Sputum	**MTB**	19.26	26.35	29.03	Positive	7.09
Urine	**MTB**	22.20	28.76	28.50	Positive	6.56
Urine	**MTB**	21.12	27.85	28.31	Positive	6.73
Biopsy	**MTB**	24.21	29.66	29.83	Positive	5.45
BAsp	**MTB**	19.78	26.10	28.14	Positive	6.32
Biopsy	**MTB**	20.94	28.00	30.18	Positive	7.06
BLav	**MTB**	21.28	27.99	29.86	Positive	6.71
Urine	**MTB**	23.57	27.44	29.91	Positive	3.87
Sputum	**MTB**	20.76	27.79	29.28	Positive	7.03
Sputum	**MTB**	22.22	29.43	28.55	Positive	7.21
Sputum	**MTB**	23.74	26.77	28.93	Positive	3.03
Sputum	**MTB**	20.93	27.83	29.33	Positive	6.9
Sputum	**MTB**	19.12	26.31	30.05	Positive	7.19
Sputum	**MTB**	19.19	25.66	28.30	Positive	6.47
Sputum	**MTB**	21.93	27.96	31.07	Positive	6.03
Sputum	**MTB**	19.81	26.97	28.54	Positive	7.16
Sputum	**MTB**	19.23	25.91	28.34	Positive	6.68
Sputum	**MTB**	17.84	24.19	28.42	Positive	6.35
Sputum	**MTB**	21.91	27.49	28.13	Positive	5.58
Sputum	**MTB**	20.48	25.55	29.03	Positive	5.07
Sputum	**MTB**	21.51	29.32	28.78	Positive	7.81
Sputum	**MTB**	16.95	21.8	28.16	Positive	4.85
Sputum	**MTB**	16.52	21.87	27.88	Positive	5.35
Sputum	**MTB**	16.69	23.22	28.35	Positive	6.53
Sputum	**MTB**	20.35	26.23	28.35	Positive	5.88
Sputum	**MTB**	20.83	27.23	28.18	Positive	6.4
Sputum	**MTB**	16.57	23.00	27.60	Positive	6.43
Sputum	**MTB**	19.81	26.02	29.20	Positive	6.21
Sputum	**MTB**	21.58	26.98	28.56	Positive	5.4
BLav	**MTB**	17.61	23.77	28.30	Positive	6.16
BLav	**MTB**	21.57	27.37	28.31	Positive	5.8
Sputum	**MTB**	20.53	25.72	30.22	Positive	5.19
	**Mean**	20.03	26.20	28.81		6.17
	**SD**	1.930	1.928	0.762		0.871

Table S1: Descriptive data of diagnostic test results obtained using the SD Biosensor Standard M10 MTB/NTM assay and the STANDARD Q TB MPT64 Ag test for the rapid identification of MTB positive cultures. Acronyms: BAsp, bronchial aspirate; BLav, bronchial lavage; LNA, Lymph Node Aspirate; CSF, Cerebrospinal fluid; PF, Pleural Fluid.

Table S2: Descriptive data of diagnostic test results obtained from NTM cultures

CULTURE MATRIX	NTM IDENTIFICATION	SD BIOSENOR RESULT	MTB Ct	NTM Ct	IC Ct	STANDARD Q TB MPT64 Ag	Δ Ct (NTM-MTB)
BAsp	***M. avium***	**NTM**	-	16.48	28.06	NEG	
BAsp	***M. avium***	**NTM**	-	19.49	28.28	NEG	
BLav	***M. simiae***	**NTM**	-	17.76	28.03	NEG	
Sputum	***M. fortuitum***	**NTM**	-	19.12	27.86	NEG	
BAsp	***M. intracellulare***	**NTM**	-	19.47	27.83	NEG	
BAsp	***M. intracellulare sub. chimaera***	**NTM**	-	15.74	27.99	NEG	
Sputum	***M. avium***	**NTM**	-	22.25	29.28	NEG	
BAsp	***M. intracellulare***	**NTM**	-	14.96	28.28	NEG	
BAsp	***M. avium***	**NTM**	-	16.31	28.44	NEG	
BLav	***M. gordonae***	**NTM**	-	18.77	28.03	NEG	
BAsp	***M. avium***	**NTM**	-	15.98	29.84	NEG	
BLav	***M. chimaera***	**NTM**	-	16.68	28.02	NEG	
Sputum	***M. avium***	**NTM**	-	18.38	28.84	NEG	
BAsp	***M. avium***	**NTM**	-	21.06	30.68	NEG	
Sputum	***M. avium***	**NTM**	-	19.9	28.62	NEG	
BAsp	***M. chelonae***	**NTM**	-	19.42	27.91	NEG	
BAsp	***M. avium***	**NTM**	-	19.18	29.51	NEG	
BLav	***M. intracellulare***	**NTM**	-	19.5	28.39	NEG	
Sputum	***M. avium***	**NTM**	-	21.49	27.96	NEG	
Sputum	***M. chimaera***	**NTM**	-	17.54	27.59	NEG	
BLav	***M. abscessus***	**NTM**	-	19.84	28.95	NEG	
Biop	***M. avium***	**NTM**	-	19.84	28.95	NEG	
BLav	***M. gordonae***	**NTM**	-	15.84	28.29	NEG	
Sputum	***M. abscessus***	**NTM**	-	19.92	29	NEG	
BLav	***M. avium complex***	**NTM**	-	17.07	28.68	NEG	
Sputum	***M. abscessus***	**NTM**	-	19.16	28.23	NEG	
BAsp	***M. gordonae***	**NTM**	-	20.93	28.32	NEG	
BLav	***M. avium***	**CO-INFECTION**	24.91	19.33	28.42	NEG	5.58
BLav	***M. chimaera***	**NTM**	-	16.97	27.87	NEG	
BAsp	***M. intracellulare***	**NTM**	-	14.94	28.44	NEG	
Sputum	***M. gordonae***	**NTM**	-	19.93	28.9	NEG	
BAsp	***M. avium***	**NTM**	-	22.06	28.46	NEG	
BLav	***M. celatum***	**NEG**	-	**37.04**	28.36	NEG	
BLav	***M. gordonae***	**NTM**	-	15.17	27.95	NEG	
BAsp	***M. avium***	**NTM**	-	18.97	27.78	NEG	
Sputum	***M. avium***	**NTM**	-	16.28	28.5	NEG	
BLav	***M. avium***	**NTM**	-	22.42	28.58	NEG	
BAsp	***M. avium***	**NTM**	-	25.67	27.98	NEG	
BAsp	***M. fortuitum***	**NTM**	-	22.22	27.85	NEG	
BAsp	***M. avium***	**NTM**	-	17.04	28.06	NEG	
Sputum	***M. intracellulare***	**NTM**	-	16.28	28.69	NEG	
BAsp	***M. intracellulare***	**NTM**	-	15.08	28.39	NEG	
BAsp	***M. mucogenicum***	**NTM**	-	22.37	27.47	NEG	
BLav	***M. intracellulare***	**NTM**	-	16.41	28.07	NEG	
BLav	***M. avium***	**NTM**	-	20.98	28.68	NEG	
Sputum	***M. gordonae***	**NTM**	-	16.8	27.75	NEG	
BAsp	***M. abscessus***	**NTM**	-	21.28	28.06	NEG	
BLav	***M. avium***	**NTM**	-	16.96	28.45	NEG	
BAsp	***M. xenopi***	**NTM**	-	18.76	28.49	NEG	
BLav	***M. chimaera***	**CO-INFECTION**	27.2	23.36	27.52	NEG	3.84
		**Mean**	26.9	19.37	27.83		4.71
		**SD**	1.765	3.718	4.0378		

Table S2: Descriptive data of diagnostic test results obtained using the SD Biosensor Standard M10 MTB/NTM assay and the STANDARD Q TB MPT64 Ag test for the rapid identification of NTB positive cultures. Acronyms: BAsp, bronchial aspirate; BLav, bronchial lavage; LNA, Lymph Node Aspirate; CSF, Cerebrospinal fluid; PF, Pleural Fluid.

Table S3: Descriptive data of diagnostic test results obtained from negative MTIG cultures

CULTURE MATRIX	SD BIOSENOR RESULT	MTB Ct	NTM Ct	IC Ct	STANDARD Q TB MPT64 Ag
Sputum	**NEG**	-	-	27.89	NEG
Sputum	**NEG**	-	-	27.54	NEG
BAsp	**NEG**	-	-	27.63	NEG
BLav	**NEG**	-	-	27.71	NEG
BLav	**NEG**	-	-	28.51	NEG
BLav	**NEG**	-	-	27.47	NEG
BLav	**NEG**	-	-	27.40	NEG
BAsp	**NEG**	-	-	28.54	NEG
BLav	**NEG**	-	-	28.61	NEG
BLav	**NEG**	-	-	27.92	NEG
BLav	**NEG**	-	-	27.60	NEG
BLav	**NEG**	-	-	28.75	NEG
BLav	**NEG**	-	-	28.42	NEG
BLav	**NEG**	-	-	28.92	NEG
BAsp	**NEG**	-	-	30.11	NEG
BLav	**NEG**	-	-	27.81	NEG
BAsp	**NEG**	-	-	28.73	NEG
BAsp	**NEG**	-	-	27.60	NEG
BAsp	**NEG**	-	-	27.74	NEG
BAsp	**NEG**	-	-	28.01	NEG
LNA	**NEG**	-	-	27.49	NEG
BAsp	**NEG**	-	-	27.47	NEG
Sputum	**NEG**	-	-	27.72	NEG
Sputum	**NEG**	-	-	27.31	NEG
Sputum	**NEG**	-	-	28.01	NEG
Sputum	**NEG**	-	-	31.07	NEG
BAsp	**NEG**	-	-	27.56	NEG
BAsp	**NEG**	-	-	28.82	NEG
BLav	**NEG**	-	-	28.42	NEG
BLav	**NEG**	-	-	28.19	NEG
BAsp	**NEG**	-	-	28.63	NEG
BAsp	**NEG**	-	-	28.83	NEG
Sputum	**NEG**	-	-	28.87	NEG
BLav	**NEG**	-	-	35.99	NEG
BLav	**NEG**	-	-	28.80	NEG
Sputum	**NEG**	-	-	28.53	NEG
BLav	**NEG**	-	-	28.90	NEG
BLav	**NEG**	-	-	29.14	NEG
BAsp	**NEG**	-	-	28.46	NEG
BLav	**NEG**	-	-	27.99	NEG
BAsp	**NEG**	-	-	28.88	NEG
BAsp	**NEG**	-	-	28.15	NEG
BAsp	**NEG**	-	-	28.42	NEG
Sputum	**NEG**	-	-	28.28	NEG
BAsp	**NEG**	-	-	30.19	NEG
BAsp	**NEG**	-	-	28.36	NEG
Sputum	**NEG**	-	-	28.19	NEG
BAsp	**NEG**	-	-	28.52	NEG
BAsp	**NEG**	-	-	28.66	NEG
BAsp	**NEG**	-	-	28.68	NEG
	**Mean**	-	-	29.09	
	**SD**	-	-	1.653	

Table S3: Descriptive data of diagnostic test results obtained using the SD Biosensor Standard M10 MTB/NTM assay and the STANDARD Q TB MPT64 Ag test on fifty negative MGIT cultures. Acronyms: BAsp, bronchial aspirate; BLav, bronchial lavage; LNA, Lymph Node Aspirate; CSF, Cerebrospinal fluid; PF, Pleural Fluid.
